# Dose-Dependent Metabolic Alterations in Human Cells Exposed to Gamma Irradiation

**DOI:** 10.1371/journal.pone.0113573

**Published:** 2014-11-24

**Authors:** Yong-Kook Kwon, In Jin Ha, Hyun-Whee Bae, Won Gyo Jang, Hyun Jin Yun, So Ra Kim, Eun Kyeong Lee, Chang-Mo Kang, Geum-Sook Hwang

**Affiliations:** 1 Integrated Metabolomics Research Group, Western Seoul Center, Korea Basic Science Institute, Seoul, 120-140, Republic of Korea; 2 Division of Radiation Effect, Korea Institute of Radiological & Medical Sciences, Seoul, 139-706, Korea; 3 Graduate School of Analytical Science and Technology, Chungnam National University, Daejeon, 305-764, Republic of Korea; National Research Council of Italy, Italy

## Abstract

Radiation exposure is a threat to public health because it causes many diseases, such as cancers and birth defects, due to genetic modification of cells. Compared with the past, a greater number of people are more frequently exposed to higher levels of radioactivity today, not least due to the increased use of diagnostic and therapeutic radiation-emitting devices. In this study, ultra-performance liquid chromatography-quadrupole time-of-flight mass spectrometry (UPLC-QTOF-MS)-based metabolic profiling was used to investigate radiation- induced metabolic changes in human fibroblasts. After exposure to 1 and 5 Gy of γ-radiation, the irradiated fibroblasts were harvested at 24, 48, and 72 h and subjected to global metabolite profiling analysis. Mass spectral peaks of cell extracts were analyzed by pattern recognition using principal component analysis (PCA) and partial least squares-discriminant analysis (PLS-DA). The results showed that the cells irradiated with 1 Gy returned to control levels at 72 h post radiation, whereas cells irradiated with 5 Gy were quite unlike the controls; therefore, cells irradiated with 1 Gy had recovered, whereas those irradiated with 5 Gy had not. Lipid and amino acid levels increased after the higher-level radiation, indicating degradation of membranes and proteins. These results suggest that MS-based metabolite profiling of γ-radiation-exposed human cells provides insight into the global metabolic alterations in these cells.

## Introduction

Recently, humans have become exposed to more radiation, both natural and artificial. Exposure to natural radiation has increased due to destructive changes in the environment, such as depletion of the atmospheric ozone layer. Moreover, exposure to artificial radiation has increased due to its use in cancer therapy, medical test equipment (such as X-ray-emitting computed tomography (CT) scanners), waste from the nuclear power industry, and nuclear accidents [1]. Excessive exposure to radiation causes serious health problems, because the effects include a series of molecular-, cellular-, and tissue-level processes [2].

The effects of exposure to radiation are complex, so have been analyzed in an overall manner. Analyses of the complex responses induced by radiation require high-throughput data processes and have depended on various “-omics” platforms, such as transcriptomics, proteomics, and metabolomics. Transcriptomics [3], [4] and proteomics [5], [6] were first used to assess the effects of ionizing radiation, followed by metabolomics [7]. Such efforts have discovered radiation-induced perturbations of RNA and protein molecules and have been successful in developing concepts such as a threshold dose [3], [5], [8]. However, any understanding of the response to radiation from such analyses is incomplete without a study of the metabolome. Analyses of the metabolome facilitate characterization of complex phenotypes, because changes in metabolites reflect systemic responses to environmental or genetic interventions, and the number of metabolites investigated is lower than the number of genes or proteins in organisms [9], [10].

A metabolomics approach as a nontarget global profiling analysis is becoming increasingly popular as a source of biomedical data using mass spectrometry (MS) and nuclear magnetic resonance (NMR). Many studies have reported biomarkers in biofluids (urine and serum) for exposure to radiation [2], [11], [12], [13]. Some metabolomics studies based on ultra-performance liquid chromatography (UPLC), and gas chromatography-mass spectrometry (GC-MS) have reported metabolites from energy, purine, and pyrimidine metabolism as urinary biomarkers of radiation exposure in rodents [14], [15], [16], [17]. It has been reported that LDL/VLDL and amino acid levels were increased in the sera of γ-irradiated mice [2]. Amino acid levels in the urine of rats were increased following radiation exposure [13]. Patterson *et al.* [7] analyzed water-soluble metabolites induced by radiation, and reported that levels of antioxidants and metabolites related to DNA synthesis were decreased.

In the present study, we investigated global metabolic responses to γ-radiation in human fibroblasts. The cells were subjected to γ-radiation doses of 1 and 5 Gy, and then harvested at 24, 48, and 72 h post irradiation. The changes in metabolites were investigated by MS-based metabolite profiling analysis. Our data suggest that the metabolic responses over time differed between cells exposed to lower (1 Gy) and higher (5 Gy)-level irradiation.

## Materials and Methods

### Chemicals and reagents

HPLC-grade water, acetonitrile, and methanol were purchased from Honeywell Burdick & Jackson (Morristown, NJ, USA), and formic acid (99% purity) was from Sigma-Aldrich (St. Louis, MO, USA).

### Cell culture, radiation exposure, and sample collection

Human primary cultured dermal fibroblasts (NF46) were purchased from Young Sciences Inc. and maintained in DMEM, supplemented with 10% fetal bovine serum (FBS), 100 U/mL penicillin, and 100 µg/mL streptomycin at 37°C in a humidified, 5% CO_2_ incubator. Cells were grown in 100-mm culture dishes to subconfluency (∼80–90%) and subcultured 1∶4. The cultured cells were exposed to γ-rays from a ^137^Cs γ-ray source at a dose rate of 2.73 Gy/min. Cells were collected at 24, 48, and 72 h after exposure to 1 and 5 Gy. Cells were harvested by trypsin-EDTA and enumerated using a TC20 Automated cell counter (Bio-Rad, Hercules, CA, USA). Harvested cells were washed with PBS once and centrifuged (500× *g*, 5 min, 4°C). Cell pellets were resuspended in 500 µL of cold DW and disrupted using an ultrasonicator (VCX-750, Sonics & Materials, Newtown, USA). The ultrasonication process was performed for 30 s at 150 W, consisting of repeated cycles of 0.5-s ultrasonication followed by 0.5-s rest. Samples were placed in an ice water bath during cell disruption. Samples were stored at −80°C until analysis.

### Cell proliferation assay, MTT assay, LDH cytotoxicity detection assay, and DCFDA assay

#### Cell proliferation assay

Cells were seeded on 12-well plates (4×10^4^cells/well) and incubated with/without treatment with γ-radiation. The TC20 Automated Cell Counter (Bio-Rad) was used to determine the cell number.

#### MTT assay

Cell viability was quantified using a colorimetric MTT assay that measures mitochondrial activity in cells. Briefly, cells seeded at a density of 4×10^4^/well in a 12-well plate (Corning, Corning, NY, USA) were allowed to adhere overnight. Before cell harvest, aliquots of MTT stock solution were added to each well, and the plate was incubated at 37°C in a humidified 5% CO_2_ incubator. After 2 h, the medium was removed and the precipitated formazan crystals were dissolved by adding 500 µL of DMSO to each well. The microplates were shaken at room temperature for 30 min and the optical density of each well was measured at 560 nm.

#### LDH cytotoxicity detection assay

LDH released into cultured media was measured using the LDH cytotoxicity detection kit (Takara Bio Inc., Otsu, Japan).

#### DCFDA assay

A flow cytometer was used to determine ROS levels. For total cells, ROS generation was measured using a fluorescence probe, DCFDA (Molecular Probes-Invitrogen, Carlsbad, CA, USA). For mitochondria-related ROS levels, mitotracker MitoSOX (Molecular Probes-Invitrogen) was used.

### Metabolite extraction

To extract metabolites from the cells, two sequential extractions, with water and 80% methanol, were conducted. The collected fibroblasts were placed in a 1.5-mL tube. Each sample was mixed with 0.5 mL of water, and vortexed for 10 s, followed by centrifugation (13,000× *g*, 10 min, 4°C). The upper layer was decanted to a fresh tube. The cell pellet was resuspended in 1 mL of methanol/water (4∶1) and vortexed for 1 min, followed by centrifugation (13,000× *g*, 10 min, 4°C). The 80% MeOH extraction was repeated. The upper layer was transferred in 500-µL aliquots to new 1.5-mL tubes. All supernatants were collected and then dried using a rotary evaporator. The dried metabolite pellets were redissolved in 450 µL of 50% MeOH and filtered through a 0.2-µm hydrophilic PTFE syringe filter (Millex, Millipore, Ireland).

### UPLC and MS analysis conditions

For analysis of the complex chemical components of human cells, an Acquity UPLC systems, including a binary solvent manager, sample manager, column heater, and photodiode array detector, was coupled with hybrid quadrupole time-of-flight tandem mass spectrometry UPLC-ESI/Q-TOF (ESI/Triple TOF 5600, AB SCIEX, Concord, ON, Canada). Chromatographic separation of samples was performed on AQUTIC UPLC HSS T3 column (2.1 mm×100 mm, 1.7 µm) at 40°C using water containing 0.1% formic acid (A) and acetonitrile containing 0.1% formic acid (B). The gradient elution program was isocratic 0.1% B for 2 min, 0.1–50% B at 2–5 min, 50–99.9% B at 5–15 min, and isocratic 99.9% B for 2 min, and then the initial conditions for 2 min. The flow rate was maintained at 0.35 mL/min.

Data acquisition was performed using a Triple TOF 5600 system with Turbo V sources and a Turbo ion spray interface. TOF MS data were acquired in positive (negative) using an ion spray voltage of 4.5 kV (−4.5 kV), nebulizer gas (Gas 1) of 50 psi, heater gas (Gas 2) of 60 psi, curtain gas (CUR) of 30 psi, a turbo spray temperature of 500°C, and declustering potential (DP) of 90 V (−90 V). MS and MS/MS data were gathered in positive mode by the UPLC-ESI/Q-TOF, a hybrid triple quadrupole time-of-flight mass spectrometer equipped with Turbo V sources and a Turbo ion spray interface. For TOF MS/MS data, information-dependent acquisition (IDA) was used with the following acquisition conditions: survey scans of 250 ms, product ion scans of 100 ms, high-resolution mode, DP of 90 V (−90 V), collision energy (CE) of 30 V (−30 V), and collision energy spread (CES) of 15 V. The TOF MS and IDA scan was operated over the mass range of *m/z* 100–1200. TOF MS and product ion calibration were performed daily in both high-sensitivity and high-resolution modes before analysis. A total of 5 µl of 80% methanol solution as a blank and pooled aliquots from each cell extract as a quality control (QC) were injected every 10 samples.

### Data processing for UPLC-QTOF MS

UPLC-ESI/Q-TOF raw data files were converted to mzML format using the AB SCIEX MS data converter software (AB SCIEX). They were preprocessed using the MZmine 2.10 software, an open-source framework for processing of mass spectrometry-based molecular profile data [18]. First, each MS spectrum was imported and filtered using the “Savitzky- Golay” algorithm. The “Baseline cut off” algorithm was used for baseline correction of the filtered data. Peak detection was performed in the following three steps: first, the individual ions above the given noise level are detected in each mass spectrum, and a mass list is generated; second, chromatograms were formed by connecting consecutive ions that could be detected continuously over the scans; and third, these chromatograms were deconvoluted into individual peaks using the “local minimum search” algorithm. An isotopic peaks grouper was used to identify isotope patterns, and then additional isotopic peaks were removed from the peak list, except for the highest isotope. To match relevant peaks in the peak lists, peak list alignment was conducted with the “Join aligner” algorithm. Following alignment, “Gap filling” was done to correct a number of occasions containing missing peaks as a product of deficient peak detection or a mistake in the alignment of different peak lists.

Median-fold-change normalization was used to remove systematic variation between samples from experiments [19]. To decrease instrumental bias, such as noise and variation, the following two processes were used to filter reliable peaks. Background peak ions in raw data were eliminated based on the ratios of peak intensities of the QC to a blank (QC/blank >3). In addition, peaks showing large variations in QC were eliminated (CV >30%).

### Identification of metabolites

Metabolites were tentatively identified by connecting to various metabolite databases, such as HMDB, MassBank, LipidMaps, and METLIN [20], [21], [22], [23]. The neutral mass was the primary term for database searching, within a tolerance. Isotopic pattern similarity can then be used as a second filter to select optimal candidates, by comparing the ratios of the detected isotopes and matching isotopes from the predicted isotopic pattern of the database compound. The fragment pattern of metabolites in experiments was compared with the corresponding fragment pattern in the databases as a third filter. Thus, the *m/z* of the precursor ion, isotope pattern matching, and fragment pattern similarity were used for the identification of the metabolites. Because MS fragmentation of each individual molecule results in a unique MS/MS fingerprint, similarities between MS fragmentations of identified MS peaks and of non-identified MS peaks were calculated to find similar metabolites to non-identified MS peaks. Similarities were determined by a modified cosine calculation that took into account the relative intensities of the fragment ions as well as the precursor *m/z* differences between the paired spectra. Cosine threshold values were set to 0.5 for similarity of non-identified and identified peaks, whereby a cosine value of 1.0 indicates identical MS/MS spectra [24].

### Data analysis

Chemometric methods were used to characterize and visualize differences and similarities among the different species. Principal component analysis (PCA) is an unsupervised method that analyzes samples in the absence of information about the samples. Prior to PCA, all variables obtained from UPLC-MS data sets were scaled to unit variance (UV). Partial least squares-discriminant analysis (PLS-DA) was used to maximize class discrimination. PCA, and PLS-DA were conducted using SIMCA-P+ (ver. 12.0; Umetrics, Umea, Sweden). *K*- means clustering was performed using the MeV software (ver. 4.9.0). The Kruskal-Wallis test (non-parametric ANOVA) and Tukey test using ranks as the *post-hoc* test of the Kruskal-Wallis test were applied using R (ver. 2. 12. 0).

## Results

Human primary cultured dermal fibroblasts (NF46) were exposed to γ-radiation and incubated for 24, 48, and 72 h. There were 10 culture dishes in each group, with the exception of one group that comprised five because five culture dishes collected 72 h after exposure to 1 Gy became contaminated during incubation. The numbers of cells in the dishes, and numbers of culture dishes, are summarized in Table S1.

### Cell proliferation assay, MTT assay, LDH cytotoxicity detection assay, and DCFDA assay

**Figure 1:**
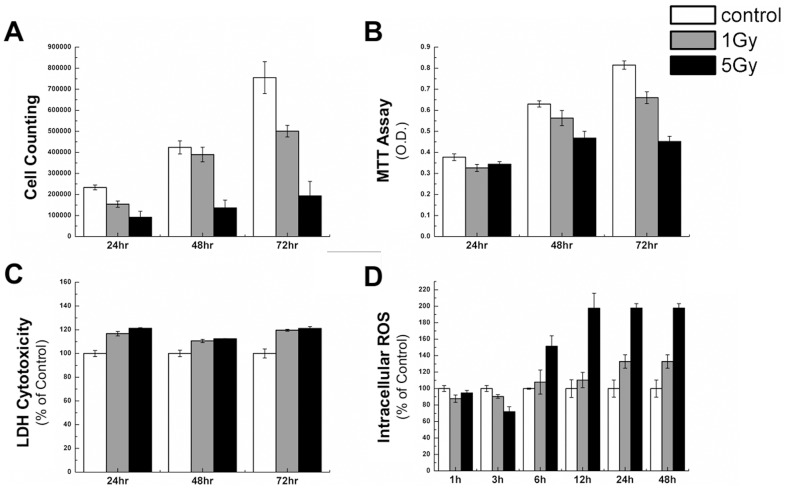
A is from the cell proliferation assay, B from the MTT assay, C from the LDH cytotoxicity detection assay, and D from the DCFDA assay. Control, 1 Gy, and 5 Gy samples for cell numbers, MTT O.D., and LDH cytotoxicity were irradiated with mock, 1 Gy, and 5 Gy at 24, 48, and 72 h post radiation, in A, B, and C respectively; Control, 1 Gy, and 5 Gy samples for intracellular ROS were irradiated with mock, 1 Gy, and 5 Gy at 1, 3, 6, 12, 24, and 48 h post radiation, in D, respectively.

Cell proliferation assay, MTT assay, LDH cytotoxicity detection assay, and DCFDA assay for total ROS levels in cells were performed (Figure 1). The results indicated that a large portion of cells maintained their viability after 72 h and only a few cells were dead after radiation exposure, which may be associated with growth retardation. Radiation may produce ROS, but this does not guarantee cell death. The effect of ROS in cells may differ according to the amount and time of exposure.

### Multivariate analysis of MS data

**Figure 2:**
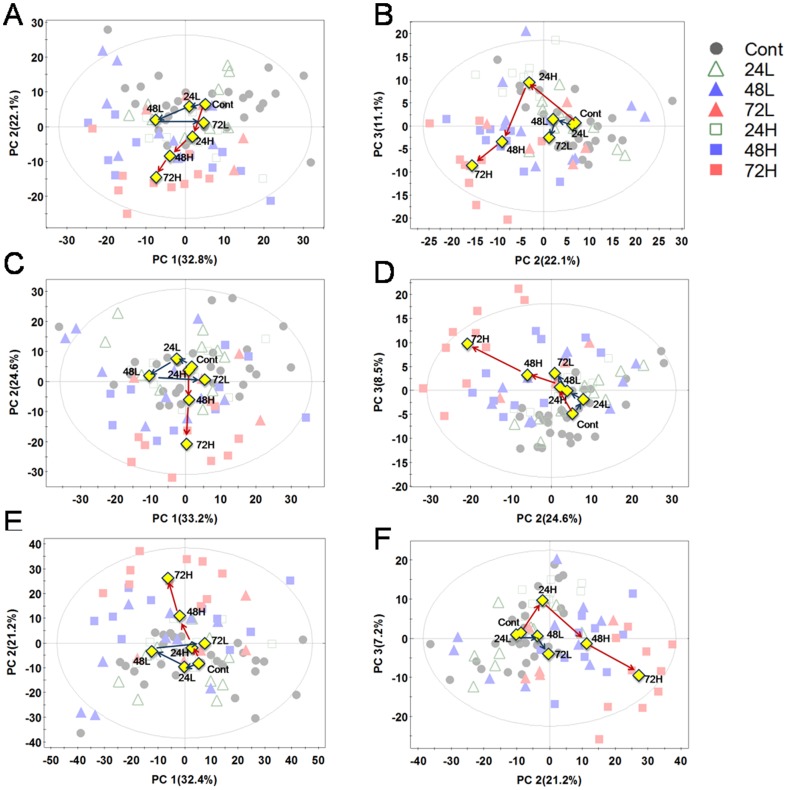
PCA score plots were derived from UPLC-QTOF-MS. A and B were from positive mode, C and D were from negative mode, and E and F were from the integration of positive and negative modes. Blue arrows and red arrows indicate the flow of cells irradiated with 1 and 5 Gy, respectively. Triangles and squares represent samples irradiated with 1 and 5 Gy, respectively. Yellow diamond marks indicate the averages of each group. Cont, control; 24L, 48L, and 72 L samples irradiated with 1 Gy at 24, 48, and 72 h post radiation, respectively.; 24H, 48H, and 72H samples irradiated with 5 Gy at 24, 48, and 72 h post radiation, respectively.

The filtered peaks in positive and negative electrospray ionization (ESI) modes were used for further analyses. Prior to performing PCA for radiation effects, only control groups were analyzed to examine changes over 3days using PCA. The Results of PCA performed in control groups showed that the control groups were mixed with other groups (Figure S1). This suggested that metabolic changes in cells did not occur by 3 days. Three groups of control cells (24, 48, 72 h) were combined as the control since we wanted to examine the effect of radiation exposure on metabolites. Peaks having large variation in the control were eliminated to remove effects of time from the control (CV >40% in the control). PCA was then used to explore changes in samples after radiation. PCA showed that irradiated groups and controls could be distinguished post irradiation. The PCA score plots from mass spectral data of cell samples collected at 24, 48, and 72 h after irradiation are shown in Figure 2. PCA score scatter plots derived from mass spectral peaks in positive and negative ion mode did not show two separate groups. However, plots using PC 1 and 2 showed characteristic patterns, whereby the sample groups irradiated with the 5-Gy dose receded from the control group whereas sample groups irradiated with 1 Gy receded from and then returned to the control group (Figure 2A, 2C). In the PCA score plot using PC 2 and 3, both groups irradiated with 1 and 5 Gy receded from the control, but the samples irradiated with 5 Gy were further from the control than those that received 1 Gy (Figure 2B, 2D). Peaks from positive and negative ion modes were integrated to investigate the comprehensive changes in cells irradiated with 1 or 5 Gy. The PCA score plot from the integrated data was very similar to the plots in each ion mode (Figure 2E, 2F). The results of the PCA score plots of data in positive and negative ion modes and the integrated data showed that the pattern of metabolites detected in positive ion mode was similar to that in negative ion mode.

**Figure 3:**
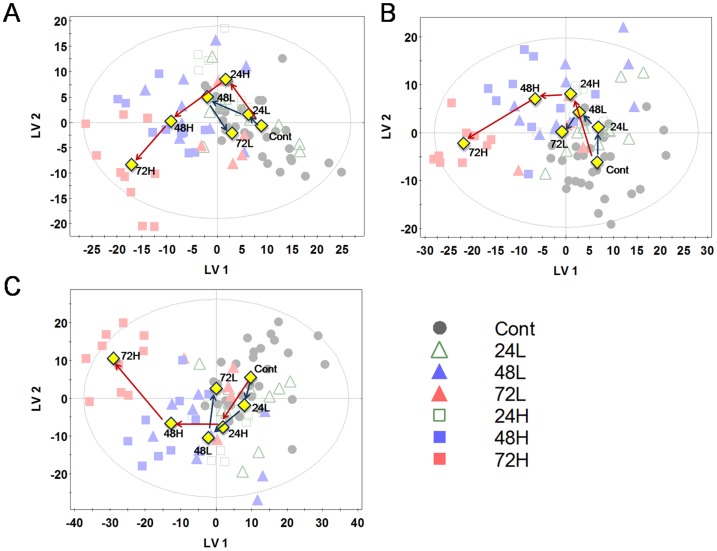
PLS-DA score plots from UPLC-QTOF-MS. A is from positive mode, B is from negative mode, C is from the integration of positive and negative modes. (A. R^2^X: 0.417, R^2^Y: 0.182, Q^2^: 0.105; B. R^2^X: 0.429, R^2^Y: 0.203, Q^2^: 0.139; C. R^2^X: 0.554, R^2^Y: 0.276, Q^2^: 0.204) Blue and red arrows indicate the flow of cells irradiated with 1 and 5 Gy, respectively. Triangles and squares indicate samples irradiated with 1 and 5 Gy, respectively. Yellow diamond marks indicate the averages of each group. Other symbols follow those in Figure 1.

The PLS-DA technique was used to maximize the metabolite pattern among the groups. PLS-DA score plots showed clearer patterns, whereby the sample groups irradiated with 5 Gy receded from the control whereas the sample groups irradiated with 1 Gy receded and then returned to the control (Figure 3). The patterns in the PLS-DA plots were similar in models generated both using data in positive and negative ion modes and integrated data using both ion modes.

### Heatmap and k-means clustering

**Figure 4:**
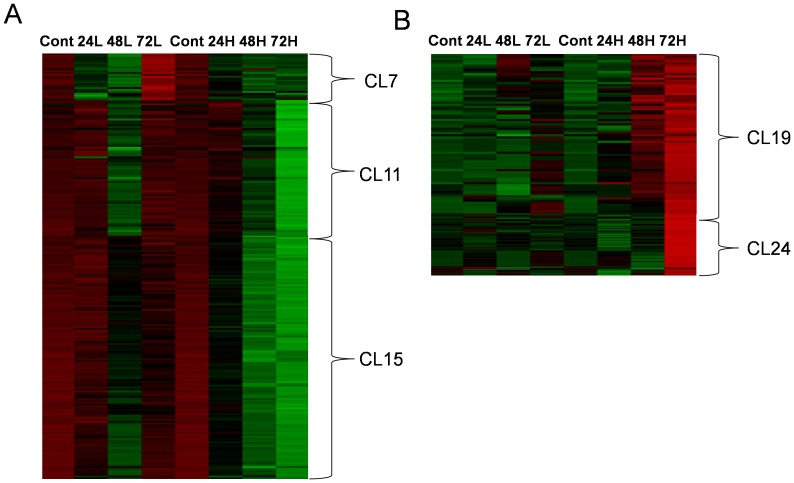
Heat map of interesting clusters from *k*-means clustering analysis. A: The clusters show the peaks that recovered from the decrease caused by radiation of 1 Gy. B: The clusters show peaks that increased 48 or 72 h after radiation with 5 Gy.

*K*-means clustering analysis was conducted to identify significant variables that affected changes in patterns in which the irradiated groups returned to control levels or receded from control groups over time (Figure S2; k = 30). The levels of the variables included in clusters 7, 11, and 15 decreased after irradiation (Figure 4A). In the samples irradiated with 1 Gy, the level of variables in clusters 7, 11, and 15 recovered in the sample collected at 72 h post radiation. However, in the samples irradiated with 5 Gy, the levels of variables in the same cluster did not recover and decreased after radiation. Clusters 7, 11, and 15 involved 25, 72, and 129 variables, respectively.

The variables in cluster 19 and 24 were increased only in samples irradiated with 5-Gy γ- radiation (Figure 4B). The variables in cluster 19 were increased in samples irradiated with 5 Gy and collected at both 48 and 72 h post irradiation. However, those in cluster 24 were increased in samples irradiated with 5 Gy and collected at 72 h post irradiation. The variables in cluster 19 and 24 were increased slightly or unchanged in the cells irradiated with 1 Gy. In total, 74 and 100 variables were involved in clusters 19 and 24, respectively.

### Identification of metabolites

Lipids (PC(14:0/0:0), PC(16:0/0:0), PC(18:1/0:0), PE(p-16:0/0:0), PE(18:1/0:0), PE(16:1/0:0), PE(20:4/0:0), PE(22:6/0:0), and arachidonic acid), amino acids (glutamic acid, tryptophan, arginine, and tyrosine), amino acid derivatives (pyroglutamic acid, cysteinylglycine, glutathione, S-acetyl-L-methionine, and alloisoleucine), choline compounds (choline, cytidine 5′-diphosphocholine, glycerophosphocholine and O-phosphocholine), nucleotide compounds (uridine diphosphate (UDP), UDP-Glc, UDP-GlcA and UDP-GalNAc, flavin adenine dinucleotide (FAD), and nicotinamide adenine dinucleotide (NAD)), and other metabolites (D-panthothenic acid, acetylneuraminic acid, justicidin B, hydroxybenzoic acid, citric acid, creatinine, and carnitinine) were identified in cell extracts using UPLC-QTOF-MS (Table 1). Relative quantification of metabolites was analyzed by using Kruskal-Wallis test to measure differences between each group. Prior to the Kruskal-Wallis test, groups were separated based on radiation dose. Identified metabolites were classified following the results of *k*-means clustering analysis (Figure S2). The quantitative information on metabolites is summarized in Table 2. We estimated putative classes of non-identified peaks through similarity of MSMS fragment patterns with identified peaks. The results are summarized in Table S2 and Table S3.

**Table 1:** List of metabolites identified from fibroblasts.

Cluster	*m/z*	R.T.	Name	Formula	Adduction	Fragments
4	146.0462	0.8	Glutamic acid	C5H9NO4	[M−H]−	102.06, 128.03,85.04
4	478.2911	10.59	PE(18:1/0:0)	C23H46NO7P	[M−H]−	281.25, 196.04,140.01
4	130.0489	0.8	Pyroglutamic acid	C5H7NO3	[M+H]+	84.04, 56.05,77.05
7	179.0476	1.19	Cysteinylglycine	C5H10N2O3S	[M+H]+	76.02, 162.02,86.99
7	218.1032	4.73	D-(+)-Pantothenic acid	C9H17NO5	[M−H]−	146.08, 88.04,71.05
7	308.0904	1.19	Glutathione	C10H17N3O6S1	[M+H]+	179.05, 162.02,76.02
9	308.0974	0.84	N-Acetylneuraminic acid	C11H19NO9	[M−H]−	87.01, 170.04,98.06
9	565.0447	0.85	UDP-Glc	C15H24N2O17P2	[M−H]−	323.03, 384.98,241.01
11	399.1434	0.84	S-Adenosyl-L-methionine	C15H22N6O5S	[M+H]+	250.09, 136.06,298.1
12	784.1465	4.73	FAD	C27H33N9O15P2	[M−H]−	437.09, 346.05,437.1
12	405.0086	0.96	UDP	C9H14N2O12P2	[M+H]+	97.03, 113.04,307.03
13	365.1046	0.89	Justicidin B	C21H16O6	[M+H]+	203.05, 185.04
13	450.2597	9.01	PE(16:10/0:0)	C21H42NO7P	[M−H]−	253.22, 196.04
16	137.0247	6.62	p-Hydroxybenzoic acid	C7H6O3	[M−H]−	93.04, 66
19	162.1118	0.81	Carnitine	C7H15NO3	[M+H]+	103.04, 85.03,60.08
19	104.1064	0.75	Choline	C5H14NO	[M]+	58.07, 60.08,59.08
19	191.0194	1.07	Citric acid, anhydrous	C6H8O7	[M−H]−	111.01, 87.01,85.03
19	132.0761	0.89	Creatine, anhydrous	C4H9N3O2	[M+H]+	90.06, 90.9,87.08
19	489.1133	0.89	Cytidine 5′-diphosphocholine	C14H26N4O11P2	[M+H]+	264.04, 184.07,360.06
19	258.1095	0.79	Glycerophosphocholine	C8H20NO6P	[M+H]+	104.11, 125,86.1
19	468.3074	7.84	PC(14:0/0:0)	C22H46NO7P	[M+H]+	184.07, 104.11,450.3
19	496.3386	9.34	PC(16:0/0:0)	C24H50NO7P	[M+H]+	184.07, 104.11,478.33
19	522.3542	9.73	PC(18:1/0:0)	C26H52NO7P	[M+H]+	184.07, 125,504.34
19	500.2755	9.43	PE(20:4/0:0)	C25H44NO7P	[M−H]−	303.23, 196.04,259.24
19	524.2755	9.39	PE(22:6/0:0)	C27H44NO7P	[M−H]−	327.23, 283.24,196.04
19	436.2808	10.63	PE(P-16:0/0:0)	C21H44NO6P	[M−H]−	196.04, 239.24,140.01
19	203.083	4.79	Tryptophan	C11H12N2O2	[M−H]−	116.05, 142.07,159.09
21	662.0985	1.04	NAD	C21H27N7O14P2	[M−H]−	540.05, 408.01,272.96
21	184.0724	0.77	O-Phosphocholine	C5H15NO4P	[M]+	125, 98.99,86.1
21	579.0238	0.87	UDP-GlcA	C15H22N2O18P2	[M−H]−	402.99, 323.03,254.99
24	303.2317	13.15	Arachidonic acid	C20H32O2	[M−H]−	259.24, 205.2
24	175.1179	0.74	Arginine	C6H14N4O2	[M+H]+	70.07, 60.06,116.07
24	130.0878	2.28	Alloisoleucine	C6H13NO2	[M−H]−	61.99, 97.93
24	164.0721	4.73	Phenylalanine	C9H11NO2	[M−H]−	147.05, 103.06,91.06
24	182.0802	2.41	Tyrosine	C9H11N1O3	[M+H]+	136.08, 91.05,123.04
24	606.0713	0.86	UDP-GalNAc	C17H27N3O17P2	[M−H]−	384.98, 282.04,402.99

**Table 2:** Relative quantification of metabolites identified in human fibroblasts.

Name	Cont	24L	48L	72L	24H	48H	72H	Post hoc (1 Gy)	Post hoc (5 Gy)
Glutamic acid#,*	4977.4±1576.9	5604.2±1654.2	6874.1±1384	7512.4±1211.8	7390.7±1691.3	8236.2±1370.3	6720.5±1735.2	c,d,f	c,e,f
PE(18:1/0:0)*	1748.3±448.1	1846.2±554.2	1751.2±408.1	1736.1±441.7	2270.3±578.7	2329.4±691.4	2149.1±486		c,e
Pyroglutamic acid*	131.9±31.2	156.8±32.9	160.2±38.3	171±33.5	177.4±32.7	206.5±23.5	180.5±46.5		c,e,f
cysteinylglycine#,*	152.4±33.5	130.9±31.5	118.3±25.9	168.7±21.9	133.1±15.3	126.2±25.8	126.9±38.1	d,e	e
D-(+)-Pantothenic acid#	6817.9±1562.6	5419.5±1218	6323.5±1270.6	7031.1±803.4	6398.4±1239.2	6241.4±796.5	6020.6±1323.1	c	
Glutathione#	38771.4±11362.7	31348.3±7502.2	32677.6±8310.1	40767.2±7453	32805±4263.2	34234.7±9631.4	33988.2±13545.1	e	
N-Acetylneuraminic acid*	1493.9±205	1410.3±171.1	1360.2±176	1381.8±199.8	1332.5±181.5	1381±347.6	1267.6±195		f
UDP-Glc#,*	13838.5±1768.8	12448.8±480.4	12695±1986.6	10664.8±1846.7	11958±2382.2	11989.5±1375.2	9663±1690.2	f	e,f
S-Adenosyl-L-methionine#,*	56.1±10.2	53.8±9.3	43.5±5.6	55.3±7.7	58.3±8	51.9±6	43.8±6.7	a,d,e	b,f
FAD#,*	431.2±63	401.9±62	383.2±54.5	347.1±39.3	372.1±38.8	349.2±69.7	422±33.5	e,f	c,d,e
UDP*	170±31.1	149.5±22.9	147.8±36.8	143.3±23.2	126±19.1	152.2±20.7	155.7±30.3		b,c
Justicidin B#,*	53.5±18.5	81±25.1	47.1±17.9	26.4±12.6	66.7±8.1	50.9±34.9	34.9±8.1	a,b,c,f	a,b,f
PE(16:1/0:0)	231.1±58.8	240.2±66.9	185.2±54.1	196.4±61.9	246.3±55.4	289.2±99.6	230.9±48.8		
p-Hydroxybenzoic acid	481.3±47.7	489.6±60.8	484.6±83.7	467.1±31.9	500.2±39.9	442.3±71.3	452.8±65.8		
Carnitine#,*	207.6±64.5	164.8±24.7	305.9±34.5	249.3±30.2	213.5±46.1	380.5±62.8	321.5±69.7	a,b,e	a,b,e,f
Choline#,*	130.3±22	135.3±17.3	220.4±42.7	171±26.6	130.1±17.8	236.5±55.7	355.3±55.6	a,b,e,f	a,b,e,f
Citric acid#	301.6±111.1	306.7±103.2	226.4±77.9	266.9±32	306.8±86.5	360.7±85.2	338±91	e	
Creatine#,*	472.5±81.4	424.7±67.7	559.1±50.7	545.1±46	456.2±73	609.2±38.3	573.8±103.4	a,b,e	a,b,e,f
Cytidine 5′-diphosphocholine#,*	39.3±10.5	60.1±12.6	39.4±16	40.4±9.6	74.4±18.9	58.4±29	83.1±18.3	a,c	a,c,d,f
Glycerophosphocholine*	519.6±112.7	649±152.8	526.2±98	470.6±107.5	546.4±103.8	660.4±84.8	777.5±179.6		b,e,f
PC(14:0/0:0)*	47.6±16.1	46.2±10.4	38.1±12	49.9±18.6	43.7±7.8	70.8±21.1	72.5±19.8		a,b,e,f
PC(16:0/0:0)*	315.6±106	329.4±80	304.2±86.1	340.4±98.1	378.3±82.4	441.4±124.2	523.6±121		b,e,f
PC(18:1/0:0)*	811.6±317.5	653±180.9	613.7±245.1	962.9±363.6	772.2±213.8	1515.9±586.9	1623.9±477.8		a,b,e,f
PE(20:4/0:0)*	1799.4±528.7	1848.7±472.9	1515.3±353	2401.9±781	2234±514.8	2797.3±865.5	3385.1±521.4		b,e,f
PE(22:6/0:0)*	1218.1±336.8	1136.3±223	1171.4±177.1	1626.9±389.8	1416.4±289	1683.1±453.1	2010.4±483.7		b,e,f
PE(p-16:0/0:0)#,*	991.2±282.8	1086.6±338.7	1488.8±340.2	1290.6±319.2	1539.5±575.5	1509.8±200.5	2188.3±472.8	e	b,c,d,e,f
Tryptophan#,*	3112.1±569.7	3028.5±552.5	3525±760.6	3858.7±333.1	3336.1±678.9	3432.7±787.9	4536.1±796	f	b,f
NAD#	1526.7±495.1	1269.3±353.5	1380.2±424.7	1588.1±286.4	1178.2±371.3	1657.5±428.8	1603.9±461.6	e,f	
O-Phosphocholine*	873.9±155.7	754.7±128.3	771.1±132.6	887.2±164.8	787.5±121.5	980.2±139.2	782.3±166.2		a,d
UDP-GlcA#,*	3777.1±1092.5	2253.5±388.1	3237±1166.2	2731.4±522.3	2548.7±421.8	3369.3±593.1	4305.9±662.6	a,c,	b,c
Arachidonic acid*	1268±399.8	1671.6±445.4	1231.4±426.3	1683.2±431.6	1825.2±510.2	1426.1±296.6	3145.2±696.9		b,c,d,f
Arginine#,*	645.7±131.9	732.6±139.4	560.7±106.4	615.1±173.2	621.1±54.9	606.8±90.7	812.5±131.8	a	b,d,f
D-Alloisoleucine*	3572.8±549.7	3356.7±880.4	3534.2±1146.4	3951.4±642.1	3399.5±534.3	3589.1±913.4	5502±822.3		b,d,f
Phenylalanine*	2804.8±368.5	2766.1±399.1	2927.8±654.7	3111±301.9	2834.4±418.1	2877.7±413.8	3837.9±571.9		b,d,f
Tyrosine*	220.6±44.4	215.7±45.5	229±40.7	234.4±40	212.8±28	212.5±27.7	308.9±58.3		b,d,f
UDP-GalNAc#,*	48299.1±7411.2	40251.3±4034.9	49700.8±5848.7	43461.7±5832.9	33892.6±2454.2	48704.9±5218.9	80952.5±9008	a,c	a,b,c,d,f

Units: peak area.

Data are given as means ± standard deviations.

Cont, control; 24L, samples irradiated with 1 Gy at 24 h post radiation; 48L, samples irradiated with 1 Gy at 48 h post radiation; 72L, samples irradiated with 1 Gy at 72 h post radiation; 24H, samples irradiated with 5 Gy at 24 h post radiation; 48H, samples irradiated with 5 Gy at 48 h post radiation; 72H, samples irradiated with 5 Gy at 72 h post radiation.

#, and * indicate significant differences between groups irradiated with 1 Gy, and 5 Gy, respectively. Letters in the *post hoc* column indicate significant differences between each group (a, 48 h vs 24 h; b, 72 h vs 48 h; c, Cont vs 24 h; d, 72 h vs 48 h; e, Cont vs 48 h; f, Cont vs 72 h) (*P*>0.05). The *P*-values were calculated by using the Kruskal-Wallis test and Tukey test using rank as *post hoc* test.

**Figure 5:**
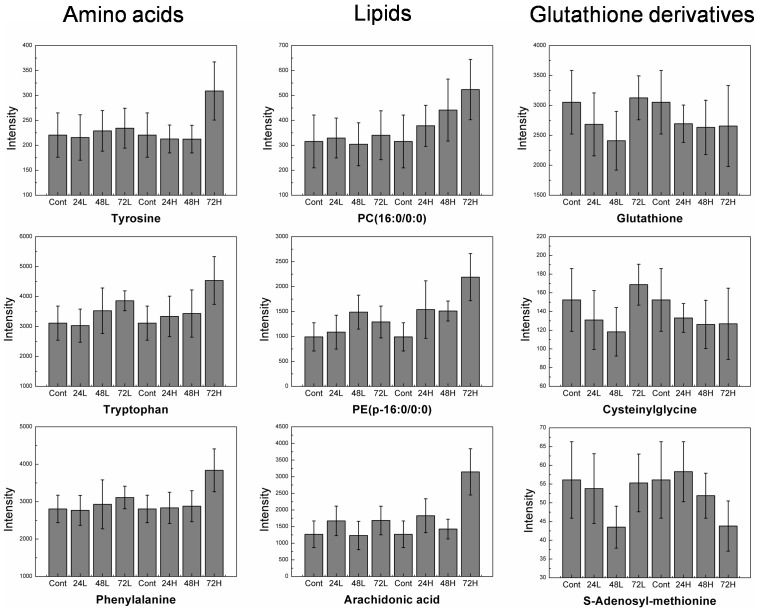
Quantification of representative metabolites of three classes (amino acids, lipids, and metabolites related to glutathione).

The metabolites in clusters 7, 11, 15, 19, and 24 in the results of the *k*-means clustering analysis were important to reveal the cellular response post irradiation. Variables in clusters 7, 11, and 15 had recovered 72 h after radiation with 1 Gy, and variables in clusters 19 and 24 increased from 48 or 72 h after radiation with 5 Gy. Glutathione, cysteinylglycine, pantothenic acid (vitamin B5), and adenosyl-methionine were included in clusters 7 and 11. Several peaks having similar MS/MS patterns to vitamin B5 were included in cluster 11. PC class, PE class, tryptophan, choline, cytidine 5′-diphosphocholine, glycerophosphocholine, citrate, creatinine, and carnitine were included in cluster 19. Arachidonic acid, arginine, alloisoleucine, phenylalanine, tyrosine, and UDP-GalNAc were included in cluster 24. Some peaks that had similar MS/MS patterns to lipids (PC class, PE class, arachidonic acid), and amino acids (tyrosine, phenylalanine, and tryptophan) were included in cluster 19. Some peaks that had similar MS/MS patterns to amino acids (tyrosine, or arginine) were included in cluster 24. Representative metabolites of specific classes such as amino acids, lipids, and glutathione derivatives are shown in Figure 5.

## Discussion

According to our results, PCA and PLS-DA showed that the cells irradiated with 5 Gy γ-radiation moved away from the controls as time passed, but those irradiated with 1 Gy γ-radiation returned to the controls as time passed. That is, upon exposure to low-level radiation (1 Gy), the cells returned to a normal state or a near-normal state rapidly. Upon exposure to higher-level radiation (5 Gy), the cells had not returned to a control state by 72 h after radiation exposure. Thus the cellular mechanisms that protect against stress were overwhelmed by exposure to higher-level γ-radiation. Although lymphoid cells such as TK6 readily undergo apoptosis after 24 h with radiation doses as low as 1.0 Gy [25], human fibroblasts exhibited a resistance to radiation in our experiment. Thus, the response to radiation differed according to cell type and species. Moreover, 1.0, 3.5, 6.5, or 8.5 Gy of total-body irradiation in nonhuman primates is equivalent to 0.76, 2.7, 5.0, and 6.5 Gy, respectively, in humans [26]. We have shown that 1 Gy induces transient metabolic changes in human fibroblasts. Since the levels of glutathione and glutathione derivatives recovered by 72 h after radiation exposure, glutathione may protect cells from damage by reducing oxygen free radical-induced oxidation. Although whether irradiation induces the same response in animal models remains unclear, analyses of the urines of irradiated mice have suggested that 0–8 Gy of gamma rays in mice [15], [16], [17], rats [14], and nonhuman primates [26] can induce systematic metabolic changes.

Glutathione, cysteinylglycine, pantothenic acid (vitamin B5), S- adenosyl-methionine, and two metabolites related to vitamin B5 decreased and then recovered in cells by 72 h after radiation with 1 Gy, but did not recover in cells irradiated with 5 Gy (Figure 5). Exposure of a cell to radiation can result in direct damage to DNA or indirect damage to genetic material through the radiolysis of water [27], [28]. Glutathione is an antioxidant that prevents damage to important cellular components caused by reactive oxygen species, such as free radicals and peroxides [29]. Cysteinylglycine is derived from the breakdown of glutathione [30], [31]. S-adenosyl-methionine is produced from adenosine triphosphate and methionine by methionine adenosyltransferase, and is an intermediate from methionine to glutathione [32]. Levels of cysteinylglycine and S-adenosyl-methionine are highly related to the level of glutathione. Changes in the levels of glutathione, cysteinylglycine, and S-adenosyl-methionine suggest that the reducing power in the cell recovered after exposure to lower-level radiation (1 Gy), but not higher-level radiation (5 Gy). Vitamin B5 is an essential metabolite for the synthesis of coenzyme-A, as well as the synthesis and metabolism of proteins, carbohydrates, and fats [33]. Aqueous vitamin B5 was degraded by free radical ions [34]. In addition, rapidly decreasing ATP and NAD in mouse leukemia cells after exposure to radiation has been reported [35]. Khan et al. [2] reported that energy metabolism in mice was disturbed after exposure to gamma-radiation. Down-regulation of energy metabolites, such as citrate and 2-oxoglutarate, in urine samples was reported in rats after irradiation [16]. Our results on changes in vitamin B5 and related metabolites levels indicate that energy metabolism was disturbed by exposure to radiation. The fibroblasts recovered from the disturbance due to lower-level (but not higher-level) radiation.

Free radical ions damaged cellular molecules and structures, including lipids, proteins, and membranes [36]. Levels of lipids (PC(18:1/0:0), PC(14:0/0:0), PC(16:0/0:0), PE(20:4/0:0), PE(22:6/0:0)), amino acids (arginine, tyrosine, phenylalanine, tryptophan), choline compounds (choline, glycerophosphocholine, cytidine 5′-diphosphocholine), and metabolites structurally related to lipids and amino acids were significantly increased in cells irradiated with 5-Gy γ-radiation and collected at 48 and 72 h or only at 72 h post irradiation (Figure 5). Membrane damage by oxidation releases phospholipids and choline compounds. Increasing levels of phospholipids and choline compounds after exposure to radiation indicated that the cell membrane was damaged by free radical ions induced by radiation. Similarly, previous studies have reported increased triacylglycerol levels in plasma due to the inhibition of lipoprotein lipase in small animals irradiated with γ-rays [37], [38]. Many earlier studies have reported the initiation of lipid peroxidation following radiation exposure [39], [40]. Khan *et al.* [2] reported that lipid and choline levels increased in the serum of γ-irradiated mice. Holecek et al. [41] described increased amino acid metabolism in rats at 48 h post irradiation with 8 Gy. Radiation induces the degeneration of skeletal muscle, resulting in the release of amino acids from irradiated muscle into the bloodstream [42].

Our results and previous studies indicate that oxygen free radical levels are increased in cells exposed to radiation (Figure 1). These free radicals damage genetic material such as DNA and RNA, and structures such as cell membranes and proteins. The lipids in membranes are degraded, increasing cytoplasmic levels of choline metabolites and lipids. Increasing intracellular amino acids levels are caused by protein degradation due to radiation and the stress response of the cell. The changes in metabolites, including glutathione, lipids, and amino acids, in cells exposed to γ-radiation indicated that increased peroxidation was increased and damage to membranes and proteins. Following exposure to lower-level radiation (1 Gy) the reduction capacity of free oxidation recovered by 72 h, and membranes and proteins exhibited little damage. However, following exposure to higher-level radiation (5 Gy) the reduction capacity of oxidation did not recover at 72 h, and damage to membranes and proteins was increased markedly. Radiation can induce molecular damages. In addition, radiation may produce ROS, although this does not guarantee cell death. The effect of ROS in cells may differ according to the amount and time of exposure. A well-known example of ROS effects is DNA double strand cleavage. However, DNA cleavage can stimulate normal cellular signaling pathways to induce the so-called “DNA damage response”. In this regard damage-induced cellular signaling may contribute to radiation-induced metabolic changes. This possibility is supported by the fact that p53 can respond to radiation-induced DNA damage and regulate various aspects of cellular metabolism [25], [43].

## Conclusions

In this study, we investigated the global metabolic responses of human cells to 1- and 5-Gy γ-radiation exposure using a UPLC-QTOF-MS-based metabolomics approach. Changes in global metabolites by radiation showed that cells irradiated with 1 Gy recovered to their normal state, but Cells treated 5 Gy did not. Metabolites associated with antioxidant and ATP synthesis recovered to control levels in cells irradiated with 1 Gy, while amino acids and lipids significantly increased in cells irradiated with 5 Gy. Upon exposure to 1-Gy γ -radiation, glutathione protected the cells from damage by reducing oxygen free radical induced-oxidation, and the levels of glutathione and glutathione derivatives recovered by 72 h after radiation exposure. However, upon exposure to 5 Gy γ-radiation, the capacity for reduction of oxygen free radicals did not recover at 72 h. ATP synthesis recovered in cells irradiated with 1 Gy, but not in cells irradiated with 5 Gy. Changes in lipids and amino acids indicated that higher radiation levels more strongly damaged proteins and cell membranes. This study provides valuable insight into the damage caused by, and recovery from, radiation exposure in terms of metabolic processes. Further studies are required to examine the response to radiation in various organisms or animal models.

## Supporting Information

Figure S1 **PCA score plots were derived from peaks of control groups: A, positive mode; B, negative mode; C, integration of positive and negative modes.** 24C, controls after 24 h; 48C, controls after 48 h; 72C, controls after 72 h.
(TIF)

Figure S2 **Total results of** ***k*** **-means clustering analysis.** Numbers in front of the heat map are number of cluster from *k*-means clustering analysis. Cont, control; 24L, samples irradiated with 1 Gy at 24 h post radiation; 48L, samples irradiated with 1 Gy at 48 h post radiation; 72L, samples irradiated with 1 Gy at 72 h post radiation; 24H, samples irradiated with 5 Gy at 24 h post radiation; 48H, samples irradiated with 5 Gy at 48 h post radiation; 72H, samples irradiated with 5 Gy at 72 h post radiation.
(TIF)

Table S1 **Summary of number of cells and number of dishes in each group.**
(DOCX)

Table S2 **Peaks associated with identified metabolites.**
(DOCX)

Table S3 **Relative quantification of peaks associated with identified metabolites.**
(DOCX)
