# Supplementary figures and images for: Dose-Dependent Metabolic Alterations in Human Cells Exposed to Gamma Irradiation
Source: PLoS One. 2014 Nov 24;9(11):e113573. doi: 10.1371/journal.pone.0113573 (PMC4242643; doi:10.1371/journal.pone.0113573)

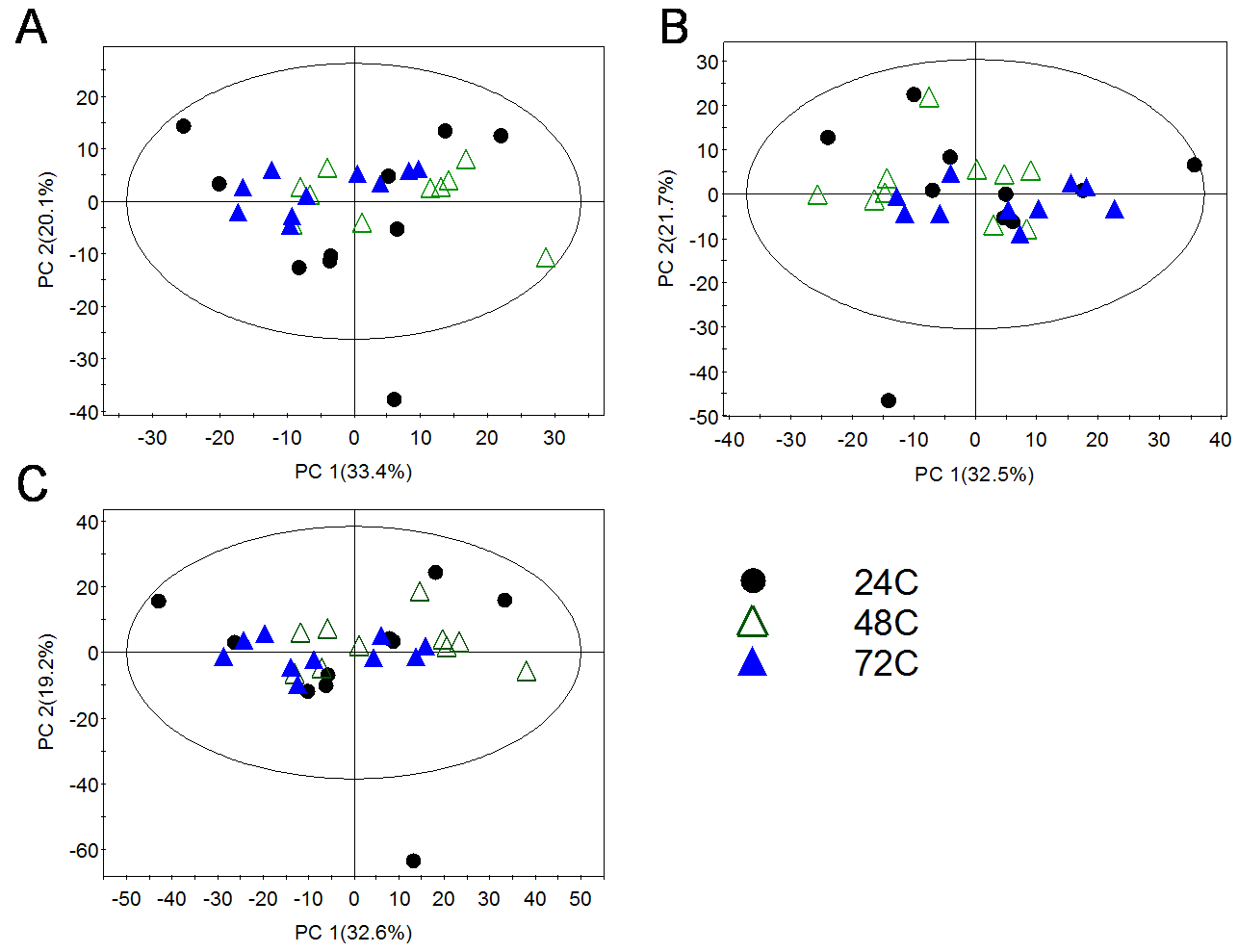

Supplement: Figure S1 — PCA score plots were derived from peaks of control groups: A, positive mode; B, negative mode; C, integration of positive and negative modes. 24C, controls after 24 h; 48C, controls after 48 h; 72C, controls after 72 h. (TIF) [file pone.0113573.s001.tif]

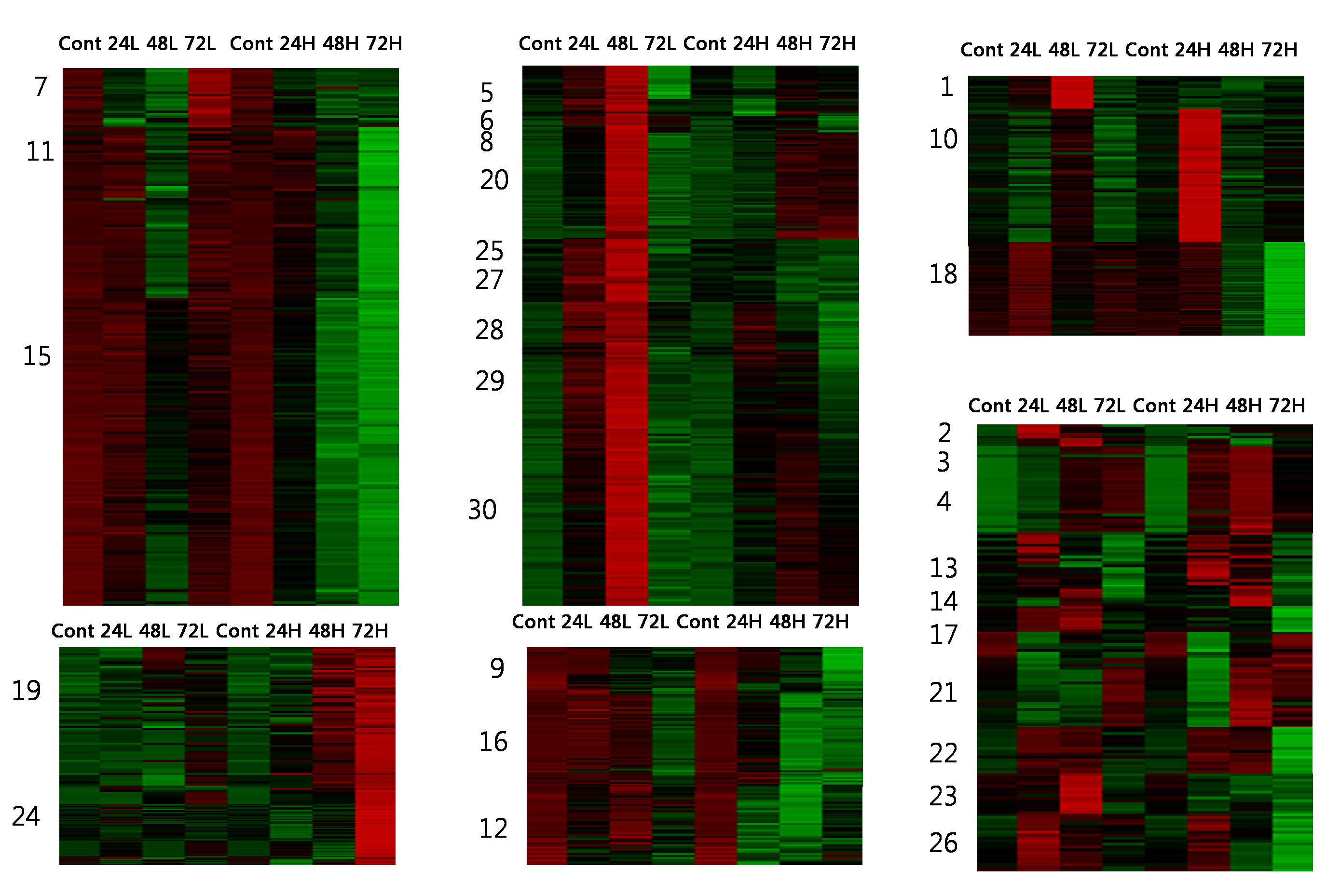

Supplement: Figure S2 — Total results of k -means clustering analysis. Numbers in front of the heat map are number of cluster from k-means clustering analysis. Cont, control; 24L, samples irradiated with 1 Gy at 24 h post radiation; 48L, samples irradiated with 1 Gy at 48 h post radiation; 72L, samples irradiated with 1 Gy at 72 h post radiation; 24H, samples irradiated with 5 Gy at 24 h post radiation; 48H, samples irradiated with 5 Gy at 48 h post radiation; 72H, samples irradiated with 5 Gy at 72 h post radiation. (TIF) [file pone.0113573.s002.tif]
