# Supplementary material for: Dose-Dependent Metabolic Alterations in Human Cells Exposed to Gamma Irradiation
Source: PLoS One. 2014 Nov 24;9(11):e113573. doi: 10.1371/journal.pone.0113573 (PMC4242643; doi:10.1371/journal.pone.0113573)
Supplement: Table S1 — Summary of number of cells and number of dishes in each group. (DOCX) [file pone.0113573.s003.docx]

**Table S1.** Summary of number of cells and number of dishes in each group.

| Radiation | Time | No. of Cells (×10^6^) | No. of Dishes |
| --- | --- | --- | --- |
| Control | 24 h | 3.22±0.4 | 10 |
|  | 48 h | 4.26±0.28 | 10 |
|  | 72 h | 3.97±0.26 | 10 |
| 1 Gy | 24 h | 1.92±0.4 | 10 |
|  | 48 h | 1.91±0.21 | 10 |
|  | 72 h | 2.06±0.04 | 5 |
| 5 Gy | 24 h | 1.27±0.16 | 10 |
|  | 48 h | 0.95±0.16 | 10 |
|  | 72 h | 1.18±0.08 | 10 |
