# Supplementary material for: Dose-Dependent Metabolic Alterations in Human Cells Exposed to Gamma Irradiation
Source: PLoS One. 2014 Nov 24;9(11):e113573. doi: 10.1371/journal.pone.0113573 (PMC4242643; doi:10.1371/journal.pone.0113573)
Supplement: Table S2 — Peaks associated with identified metabolites. (DOCX) [file pone.0113573.s004.docx]

**Table S2.** Peaks associated with identified metabolites.

| ID | Cluster | *m/z* | RT | Cosine value | Related metabolite | Mode | Fragment |
| --- | --- | --- | --- | --- | --- | --- | --- |
| Ne_370 | 4 | 204.00431 | 0.80 | 0.741 | Glutamic acid | Negative | 146.05, 128.04, 166.84 |
| Ne_400 | 4 | 128.03543 | 0.80 | 0.662 | D-Alloisoleucine | Negative | 126.02, 124.91, 120.07 |
| Po_77 | 7 | 308.09112 | 1.68 | 0.897 | Glutathione | Positive | 179.05, 162.02, 84.05 |
| Ne_696 | 7 | 277.12182 | 4.82 | 0.560 | D-(+)-Pantothenic acid | Negative | 146.08, 217.12, 71.05 |
| Ne_396 | 11 | 286.09076 | 4.70 | 0.842 | D-(+)-Pantothenic acid | Negative | 218.1, 88.04, 146.08 |
| Po_1316 | 19 | 428.17623 | 0.92 | 0.522 | Tyrosine | Positive | 136.06, 120.07, 230.1 |
| Po_2686 | 19 | 492.30775 | 7.95 | 0.604 | PC(18:1/0:0) | Positive | 184.07, 474.29, 104.11 |
| Ne_471 | 19 | 568.26445 | 9.13 | 0.580 | PE(20:4/0:0) | Negative | 500.28, 303.23, 481.23 |
| Po_1807 | 19 | 492.30775 | 7.95 | 0.604 | PC(18:1/0:0) | Positive | 184.07, 474.29, 104.11 |
| Ne_1240 | 19 | 504.26964 | 10.47 | 0.969 | PE(p-16:0/0:0) | Negative | 436.28, 196.04, 239.24 |
| Ne_1231 | 19 | 500.27464 | 9.48 | 0.569 | Arachidonic acid | Negative | 303.23, 196.04, 259.24 |
| Ne_268 | 19 | 232.05965 | 4.68 | 0.856 | L-(-)-Phenylalanine | Negative | 164.07, 147.05, 103.06 |
| Ne_336 | 19 | 271.07027 | 4.73 | 0.797 | Tryptophan | Negative | 203.08, 225.06, 116.05 |
| Ne_436 | 21 | 540.05149 | 1.05 | 0.970 | NAD | Negative | 272.96, 328.04, 158.93 |
| Ne_2113 | 21 | 760.07765 | 0.87 | 0.912 | NAD | Negative | 540.05, 638.03, 328.04 |
| Ne_530 | 21 | 698.07552 | 0.87 | 0.991 | NAD | Negative | 540.05, 328.05, 576.03 |
| Po_601 | 24 | 182.08071 | 2.88 | 0.971 | Tyrosine | Positive | 136.08, 91.05, 123.04 |
| Po_962 | 24 | 203.15001 | 0.88 | 0.500 | Arginine | Positive | 70.07, 158.13, 71.06 |
