# Supplementary material for: Dose-Dependent Metabolic Alterations in Human Cells Exposed to Gamma Irradiation
Source: PLoS One. 2014 Nov 24;9(11):e113573. doi: 10.1371/journal.pone.0113573 (PMC4242643; doi:10.1371/journal.pone.0113573)
Supplement: Table S3 — Relative quantification of peaks associated with identified metabolites. (DOCX) [file pone.0113573.s005.docx]

**Table S3.** Relative quantification of peaks associated with identified metabolites.

| ID | Cont | 24L | 48L | 72L | 24H | 48H | 72H | Post hoc  (1 Gy) | Post hoc  (5 Gy) |
| --- | --- | --- | --- | --- | --- | --- | --- | --- | --- |
| Ne_370 ^#,^* | 949.4±285.2 | 1088.3±322.1 | 1340.9±287.2 | 1465.9±210 | 1384.1±343.2 | 1623.2±234.2 | 1300.9±276.6 | e,f | c,e,f |
| Ne_400* | 921.8±294.9 | 999.1±322 | 1280.7±292.2 | 1418.2±197.5 | 1419±364.4 | 1538.2±251.9 | 1245±319.1 |  | c,e,f |
| Po_77 ^#,^* | 1920.8±375.8 | 1691±387 | 1527.2±319.6 | 2038.4±270.1 | 1636.6±202.6 | 1557.8±289.4 | 1605±458.3 | e | e |
| Ne_696 * | 242.2±94.3 | 189.3±94.7 | 156±104.7 | 271.3±141.4 | 178.3±81.8 | 131.2±81 | 201.3±64.6 |  | e |
| Ne_396 ^#,^* | 616.7±122.2 | 511.7±97.3 | 594.2±83.6 | 651±45 | 622.6±125.8 | 633±93.1 | 464.3±78 | e | d,f |
| Po_1316 ^#,^* | 37±9.6 | 49.7±9.4 | 70±9.5 | 55.5±9.2 | 43.3±3.3 | 73.9±8.5 | 121.8±10.6 | a,c,e,f | a,b,c,e,f |
| Po_2686 ^#,^* | 28.7±9.3 | 25.2±6.6 | 22.2±6.7 | 42.7±18.8 | 23.2±5.8 | 54.4±16.6 | 61.1±20 | e,f | a,b,e,f |
| Ne_471* | 498.3±187.1 | 470±125.6 | 374.8±90.3 | 610.2±196.6 | 474.6±101.9 | 815.7±269.9 | 846.9±204.6 |  | a,b,e,f |
| Po_1807 ^#,^* | 27.9±8.4 | 23.5±4.9 | 21.4±5.3 | 41.1±20.4 | 23.4±4.4 | 55.8±18.1 | 69±24.8 | d,e | a,b,e,f |
| Ne_1240 ^#,^* | 135.4±32.5 | 162±59.1 | 229.8±55.4 | 196.8±54.4 | 236±86.8 | 199±61 | 323.8±59.9 | a,e | b,c,d,e,f |
| Ne_1231* | 1801.9±529.9 | 1854.4±477.8 | 1522.9±349.7 | 2394.2±769.2 | 2238.3±515.1 | 2804.7±869.5 | 3386.4±522.2 |  | b,e,f |
| Ne_268* | 1041±116 | 1046.4±117.2 | 1098±218.7 | 1191.7±70.5 | 1082.3±139.6 | 1121±138.6 | 1302±156.6 |  | f |
| Ne_336 ^#,^* | 1131.6±190.1 | 1127.9±192.1 | 1280.2±264.5 | 1380.9±104.1 | 1221.3±225.3 | 1253.1±283.7 | 1588±243.2 | f | b,f |
| Ne_436^#^ | 829.2±300.2 | 656.8±190.8 | 752.6±216.1 | 879.3±137.6 | 625±218.3 | 890.5±228.9 | 886.2±263.9 | d,e |  |
| Ne_2113* | 912.3±336.4 | 763.4±272.1 | 739.6±300.7 | 1071.1±243.3 | 680.8±288.7 | 1043.9±323.9 | 1026.4±344.1 |  | a,b |
| Ne_530 ^#,^* | 698.7±264.8 | 672±219.5 | 594.2±250.2 | 898.9±223 | 547.2±212.4 | 961.6±279.4 | 870.1±291.3 | a,b,c,f | a,b,c |
| Po_601* | 351.4±63.1 | 350.6±51.3 | 372.3±63.9 | 373.3±71.3 | 341±33.7 | 348.2±40.5 | 493.5±79.6 |  | b,d,f |
| Po_962 ^#,^* | 196.6±70.2 | 222.9±63.4 | 142.2±32 | 170.7±49.8 | 171.6±23.6 | 114.9±21.4 | 232±42.5 | a,e | a,b,d,e,f |

Units : Peak area

Data are means ± standard deviations.

Cont, control; 24L, samples irradiated with 1 Gy at 24 h post radiation; 48L, samples irradiated with 1 Gy at 48 h post radiation; 72L, samples irradiated with 1 Gy at 72 h post radiation; 24H, samples irradiated with 5 Gy at 24 h post radiation; 48H, samples irradiated with 5 Gy at 48 h post radiation; 72H, samples irradiated with 5 Gy at 72 h post radiation.

#, and * indicate significant differences between groups irradiated with 1 Gy, and 5 Gy, respectively. Letters in the post hoc column indicate significant differences between each group (a, 48 h vs 24 h; b, 72 h vs 48 h; c, Cont vs 24 h; d, 72 h vs 48 h; e, Cont vs 48 h; f, Cont vs 72 h) (P > 0.05). The P-values were calculated by using the Kruskal-Wallis test and Tukey test using rank as post hoc test.
